# Supplementary material for: Collaborative intelligence in AI: Evaluating the performance of a council of AIs on the USMLE
Source: PLOS Digit Health. 2025 Oct 9;4(10):e0000787. doi: 10.1371/journal.pdig.0000787 (PMC12510544; doi:10.1371/journal.pdig.0000787)
Supplement: S2 File — (ZIP) [file pdig.0000787.s002.zip › S2 Data Raw data files containing unprocessed question inputs and LLM outputs including transcripts of deliberations/STEP3_Question-133-1_Type-MC-NJ.docx]

**Facilitator** **[Round 1]:** A 45-year-old man comes to the emergency department because of a 1-month history of fatigue, generalized muscle weakness, and a 4-kg (8-lb) weight loss. He also reports a loss of appetite and nausea without vomiting during the past 2 weeks. Medical history is unremarkable and he takes no medications. He does not smoke cigarettes or drink alcoholic beverages. He is 180 cm (5 ft 11 in) tall and weighs 70 kg (155 lb); BMI is 22 kg/m². Vital signs are temperature 36.4°C (97.5°F), pulse 100/min, respirations 20/min, and blood pressure 88/50 mm Hg. Physical examination shows bronze darkening of the elbows, on the creases of his hands, and around the areolae of his nipples. Muscle strength is 4.5/5 in all extremities. The remainder of the physical examination shows no abnormalities. Results of laboratory studies are shown: Serum Na⁺ 128 mEq/L K⁺ 5.2 mEq/L Cl⁻ 95 mEq/L HCO3⁻ 20 mEq/L Blood Hemoglobin 12 g/dL WBC 3500/mm³ In addition to administering intravenous fluids, which of the following is the most appropriate pharmacotherapy? (A) Calcitonin (B) Dehydroepiandrosterone (C) Hydrocortisone (D) Levothyroxine (E) Mitotane

**AI_A:** (1) RESTATING THE QUESTION: The question essentially asks about the most appropriate drug treatment for a 45-year-old male patient who presents with symptoms like fatigue, muscle weakness, weight loss, appetite loss, and nausea. This patient's physical examination shows bronze darkening of certain areas, slightly diminished muscle strength, and lab results indicating mild hyponatremia (low serum sodium), mild hyperkalemia (high serum potassium), and a slightly decreased white blood cell count. In pathophysiology, the terms hyponatremia and hyperkalemia refer to the abnormal levels of sodium and potassium ions in the bloodstream, respectively, which can impact cellular functions and the body's overall fluid balance. (2) PLANNING: The chief underlying assumptions include considering the patient's age, gender, symptoms, physical examination, and lab results. We could question whether this patient's symptoms are related to an endocrine disorder, such as Addison's disease, given the bronze darkening (hyperpigmentation), muscle weakness, fatigue, weight loss, hyponatremia, and hyperkalemia are keys features. It's also important to debate if the assumption that the issue could be caused by the adrenal gland's reduced production of cortisol and aldosterone, hence leading us to hypothesize that cortisol replacement therapy should be explored. (3) CLINICAL REASONING: Addison's disease, also known as primary adrenal insufficiency, occurs when the adrenal glands fail to produce adequate amounts of cortisol and aldosterone. This causes hyponatremia and hyperkalemia, due to the lack of aldosterone's role in maintaining sodium and potassium balance. The hyperpigmentation results from increased adrenocorticotropic hormone (ACTH) due to a lack of negative feedback from diminished cortisol. In response to hyponatremia and hyperkalemia, the body may show symptoms like muscle weakness, fatigue, and hypotension (low blood pressure). Additionally, the lack of cortisol, which aids in stress response and has anti-inflammatory effects, may cause symptoms like weight loss, loss of appetite, and fatigue. (4) CONCLUDING: Given that this patient's symptoms correlate with Addison's disease, cortisol replacement therapy would be an appropriate pharmacological intervention. This is because cortisol replacement therapy would restore the missing hormone, reducing the symptoms, and helping manage the disease. (5) RESPONSE: Therefore, among the given options, Hydrocortisone (C), a synthetic form of cortisol, would be the most appropriate pharmacotherapy for this patient.

**AI_C:** RESTATING THE QUESTION: A middle-aged male patient is presenting to the emergency department with symptoms that he has been experiencing for the last month, which include fatigue, muscle weakness, weight loss, loss of appetite, and nausea. His physical examination shows a decrease in muscle strength and a bronze-darkened skin, particularly in certain areas like the elbows, hand creases, and around the nipples. His blood panel shows hyponatremia, a slightly high potassium level, normal levels of chloride and bicarbonate. His hemoglobin and white blood cell counts are within normal limits albeit on the lower side. The query is about the choice of medication to supplement intravenous fluids in treating this patient's condition. PLANNING: The symptoms this patient is experiencing along can be attributed to various conditions ranging from malnutrition due to loss of appetite, to endocrine disorders like adrenal insufficiency or thyroid disorders. The physical features of skin darkening further direct the suspicion towards disorders like Addison's disease, which is a type of primary adrenal insufficiency. This is supported by the laboratory results, which demonstrate hyponatremia and mild hyperkalemia, commonly associated with Addison's disease due to reduced aldosterone secretion. Therefore, if we prioritize the hypothesis of adrenal insufficiency, the most suitable pharmacotherapy would be one that substitutes the deficient adrenal cortical hormones. CLINICAL REASONING: In conditions like Addison's disease, where there is an insufficiency of adrenal hormones, we can expect characteristic symptoms such as fatigue, muscle weakness, weight loss, loss of appetite, and nausea. This is due to the lack of cortisol, which is involved in the body's stress response, and aldosterone, which regulates the body's salt and water balance. The skin changes described in this case, such as the darkening in certain areas, can also be typical features of this condition due to an overproduction of melanocyte-stimulating hormone (MSH) as a result of an increased secretion of Adrenocorticotropic hormone (ACTH) in an attempt to stimulate the underactive adrenal gland. Connecting this clinical picture with the lab results, which show hyponatremia and mild hyperkalemia, the diagnosis of adrenal insufficiency seems very likely since these electrolyte imbalances would be consistent with the deficiency of aldosterone. CONCLUDING: Based on the likely diagnosis of this patient's condition as adrenal insufficiency, choosing a treatment approach involves replacing the deficient hormones. Among the choices given, hydrocortisone would be the appropriate choice as it can act as a substitute for the deficient cortisol. RESPONSE: Therefore, the most appropriate pharmacotherapy for this patient, in addition to administering intravenous fluids, would be Hydrocortisone (Option C).

**AI_D:** RESTATING THE QUESTION: This question presents a 45-year-old male with a constellation of symptoms that include fatigue, muscle weakness, weight loss, loss of appetite, and nausea. Notably, he presents with unusual physical findings, such as the bronze darkening of his elbows, hand creases, and areolae. His laboratory results reveal hyponatremia, hyperkalemia, and mild anemia. The question asks what the most appropriate pharmacotherapy would be for this patient in addition to intravenous fluids. PLANNING: Firstly, bridging his clinical symptoms with the laboratory values and vital signs, we observe signs of chronic disease with weight loss, hypotension, hyponatremia, and hyperkalemia. Furthermore, the bronzing of the skin might point to hyperpigmentation, a sign often associated with adrenal insufficiency or Addison's disease. The alternate assumption is that he might suffer from another common disease with similar presentation like hypothyroidism. However, the combined hyperpigmentation and electrolyte imbalance leans strongly in favor of the adrenal insufficiency hypothesis. CLINICAL REASONING: Adrenal insufficiency, also known as Addison's disease, is a disorder where the adrenal glands are unable to produce enough steroid hormones (mainly cortisol, but also aldosterone to a lesser extent). The fatigue, muscle weakness, weight loss, loss of appetite, and nausea are all symptoms often seen in adrenal insufficiency due to cortisol deficiency. The hyperpigmentation encountered in the patient is caused by increased production of melanocyte-stimulating hormone (MSH) secondary to elevated levels of adrenocorticotropic hormone (ACTH). This occurs due to the lack of negative feedback on the release of corticotropin-releasing hormone (CRH) and ACTH when cortisol levels are low. The hyponatremia and hyperkalemia suggest aldosterone deficiency, as this hormone is responsible for sodium reabsorption and potassium excretion in the kidneys. CONCLUDING: Considering these clinical and laboratory findings, the patient's adrenal insufficiency is most likely the result of a primary issue with the adrenal glands themselves rather than a secondary deficiency from pituitary or hypothalamic causes. Therefore, the best course of action is to replace the deficient hormones. RESPONSE: The most appropriate choice of pharmacotherapy, in this case, would be hydrocortisone (Option C), as it is a synthetic glucocorticoid that can replace the deficient cortisol and, to a lesser extent, aldosterone. Other choices like calcitonin, dehydroepiandrosterone, levothyroxine, and mitotane do not adequately address the multiple dimensions of this patient's adrenal insufficiency.

**AI_B:** RESTATING THE QUESTION: This query is about a 45-year-old male who has been experiencing symptoms such as fatigue, muscle weakness, weight loss, loss of appetite, and nausea for the past 2 weeks to 1 month. On examination, darkening of the skin at certain spots, reduced blood pressure, and alterations in serum components as well as in blood counts are identified. The query seeks to determine which pharmacological intervention would be the most appropriate, alongside administering intravenous fluids. PLANNING: Given the symptoms, we can assume that these might be associated with certain systemic conditions. These could include hormonal imbalance such as Addison's disease, thyroid disorders, or some malignancies. Addison’s disease, or primary adrenal insufficiency, occurs due to the insufficient secretion of glucocorticoids (cortisol) and mineralocorticoids (aldosterone) by the adrenal glands. In contrast, some malignancies can cause similar symptoms and lab findings due to the secretion of hormones similar in function to adrenal hormones. Therefore, we need to clinically prioritize the assumption of Addison’s Disease given the constellation of clinical and laboratory findings such as hyperpigmentation in the skin folds and areolae, hyponatremia, hyperkalemia, and hypotension. These could all point towards adrenal insufficiency. CLINICAL REASONING: In Addison's disease, the adrenal cortex is affected leading to a decrease in secretion of hormones cortisol and aldosterone. This decline in corticosteroid levels in the body gives rise to the clinical picture of tiredness, muscle weakness, weight loss, changes in skin color (hyperpigmentation), low blood pressure, and alterations in serum levels of Na⁺, K⁺, and Cl⁻. The decrease in cortisol, which is a glucocorticoid, results in the body's inability to regulate responses to stress and immune function, leading to symptoms of weakness, fatigue, and weight loss. Hyperpigmentation occurs due to elevated levels of adrenocorticotropic hormone (ACTH), since there's no negative feedback inhibition from cortisol. Increase in ACTH, in turn, leads to overproduction of melanocyte-stimulating hormone (MSH), leading to the bronzing of the skin. Low aldosterone, a mineralocorticoid, impacts the balance of electrolytes in the body leading to increased serum K⁺ and decreased Na⁺ and Cl⁻, which further lead to the symptoms of hypotension and muscle weakness. CONCLUDING: After considering the clinical symptoms, physical examination findings, and lab results, as well as understanding their underlying pathophysiology, we can connect the symptomatology of this patient to the reduction of cortisol and aldosterone due to a suspected adrenal insufficiency. For such cases, it is crucial to replenish the depleted hormones, hence considering the options provided, the administration of Hydrocortisone - which is a corticosteroid providing both glucocorticoid and minor mineralocorticoid activity – would be most appropriate. RESPONSE: Thus, the most appropriate pharmacotherapy for the described patient, along with administering intravenous fluids, would be Hydrocortisone (Option C), considering the suspected diagnosis of Addison's disease presenting as adrenal insufficiency.

**AI_E:** RESTATING THE QUESTION: This scenario presents a 45-year-old man with symptoms of fatigue, muscle weakness, weight loss, loss of appetite, and nausea over the past month. His examination notes include a bronzed complexion and laboratory results hint toward certain imbalances. The question seeks the most appropriate pharmacotherapy for this patient aside from intravenous rehydration. Let's decipher each of the provided options: (A) Calcitonin - primarily utilized for conditions related to hypercalcemia and osteoporosis, (B) Dehydroepiandrosterone (DHEA) - a steroid hormone precursor which finds use in several conditions including adrenal insufficiency, (C) Hydrocortisone - a glucocorticoid used in conditions of adrenal insufficiency, (D) Levothyroxine - for hypothyroidism, and (E) Mitotane - used mainly for adrenocortical carcinoma. PLANNING: The patient’s symptoms along with the physical findings and the lab results suggest a condition that might involve adrenal insufficiency leading to electrolyte imbalances and skin changes. His hyponatremia and hyperkalemia are typical of adrenal insufficiency. The skin pigmentation could be due to increased production of melanocyte-stimulating hormone (MSH) secondary to increased Pro-opiomelanocortin (POMC) production as a body's attempt to produce more ACTH in case of primary adrenal insufficiency. This condition, primary adrenal insufficiency is also known as Addison's disease. CLINICAL REASONING: With regard to the skin changes, Addison's disease causes an increase in the production of melanocyte-stimulating hormone (MSH), leading to hyperpigmentation, which is visible most notably on exposed areas, in skin folds, and on the nipples and areolae. The patient is showing signs of hyponatremia (low sodium), hyperkalemia (elevated potassium), and less bicarbonate than normal, all of which imply a deficiency in aldosterone, one of the hormones produced by the adrenal glands, which are damaged in Addison's disease. The weakened muscles and other symptoms are indicative of lack of cortisol, another adrenal hormone, signaling that the adrenal insufficiency might be of primary origin. CONCLUDING: Given the observed reduced sodium, increased potassium, low bicarbonate levels, and hyperpigmented skin, the patient's symptoms and signs indicate primary adrenal insufficiency (Addison's Disease), which is typically caused by autoimmune destruction of the adrenal cortex in developed countries. As such, with the lack of cortisol and aldosterone that are characteristic of Addison's disease, a synthetic replacement for these hormones would be most beneficial. One suitable treatment option is hydrocortisone, which is a synthetic glucocorticoid that also has some mineralocorticoid activity. RESPONSE: In the light of the symptoms presented, the bloodwork and physical findings, the patient's condition suggests a diagnosis of primary adrenal insufficiency (Addison's disease). To manage his condition, in addition to fluids, the most appropriate choice of pharmacotherapy would be Hydrocortisone (Option C), since it acts as a substitute for the deficient cortisol and has some aldosterone-like activity. This would correct the hormone deficiency and reverse his symptoms.

**Facilitator** The question presents a 45-year-old male patient with symptoms such as fatigue, muscle weakness, weight loss, loss of appetite, and nausea. His physical examination reveals bronze darkening of certain areas and slightly diminished muscle strength. Laboratory results indicate hyponatremia, hyperkalemia, and a slightly decreased white blood cell count. These symptoms, physical findings, and lab results suggest a condition that might involve adrenal insufficiency, possibly Addison's disease, which is characterized by the adrenal glands' failure to produce adequate amounts of cortisol and aldosterone. This leads to hyponatremia and hyperkalemia due to the lack of aldosterone's role in maintaining sodium and potassium balance. The hyperpigmentation results from increased adrenocorticotropic hormone (ACTH) due to a lack of negative feedback from diminished cortisol. The body's response to these imbalances may manifest as muscle weakness, fatigue, and hypotension. The lack of cortisol, which aids in stress response and has anti-inflammatory effects, may cause symptoms like weight loss, loss of appetite, and fatigue. Given these findings, the most appropriate pharmacotherapy for this patient, in addition to administering intravenous fluids, would be Hydrocortisone (Option C). Hydrocortisone is a synthetic glucocorticoid that can replace the deficient cortisol and, to a lesser extent, aldosterone, thereby reducing the symptoms and helping manage the disease.
